# Joint Associations of Maternal Smoking and Pre-Pregnancy BMI with Term Low Birthweight and Small-for-Gestational-Age Births: A Cross-Sectional Analysis of 2024 United States Birth Data

**DOI:** 10.3390/ijerph23070885

**Published:** 2026-07-09

**Authors:** Anthony J. Kondracki, Wei Li, Ying Sun

**Affiliations:** 1 Department of Community Medicine, Mercer University School of Medicine, Savannah, GA 31404, USA; 2 Department of Psychiatry, Yale University School of Medicine, New Haven, CT 06511, USA; wei.vanness.li@yale.edu; 3 Department of Maternal, Child & Adolescent Health, School of Public Health, Anhui Medical University, Hefei 230032, China; yingsun@ahmu.edu.cn

**Keywords:** term LBW and SGA, maternal smoking, pre-pregnancy BMI, joint effects

## Abstract

**Highlights:**

**Public health relevance—How does this work relate to a public health issue?**

- Low birthweight (LBW) represents a critical public health challenge because it determines immediate infant survival and long-term health status. Rates of low birthweight (less than 2500 g) in the United States remain high and persistent.
- This study focuses on LBW and small-for-gestational age (SGA) infants at term (37–41 weeks gestation) who appear clinically healthy, but face potential immediate and long-term health risks.

**Public health significance—Why is this work of significance to public health?**

- Smoking during pregnancy has been declining over the past two decades, and maternal pre-pregnancy body mass index (BMI) is rapidly rising.
- These are modifiable risk factors impacting newborn growth and birth weight, placing a significant financial and emotional burden on families and society.

**Public health implications—What are the key implications or messages for practitioners, policy makers and/or researchers in public health?**

- Increasing rates of LBW and pre-pregnancy BMI signal a potential future rise in chronic health issues at the population level.
- Public health efforts emphasize smoking cessation and weight reduction among women entering pregnancy to reduce risks and to improve birth outcomes.

**Abstract:**

Low birthweight (LBW; <2500 g) remains a persistent public health concern in the United States, with important implications for infant survival and long-term health. Maternal smoking and pre-pregnancy body mass index (BMI) are key determinants of fetal growth, yet their joint effects in term infants are not well characterized. Our analysis of the 2024 U.S. National Center for Health Statistics (NCHS) birth data restricted to 2,973,679 singleton term births (37–41 weeks) reveals that 2.8% of infants were born LBW and 8.1% small-for-gestational-age (SGA). Smoking during pregnancy (2.2%) was associated with higher odds of LBW (aOR 2.19; 95% CI: 1.92, 2.51), SGA (aOR 1.90; 95% CI: 1.85, 1.96), and combined SGA/LBW (aOR 2.75; 95% CI: 2.66, 2.84) than nonsmoking. Underweight BMI was associated with higher odds of SGA/LBW (aOR 1.95; 95% CI: 1.89, 2.01) and overweight/obesity with lower odds of SGA (aOR 0.69; 95% CI: 0.68, 0.70) than normal BMI. Joint associations of BMI and smoking on term SGA/LBW were strongest (aOR 5.14; 95% CI: 4.62, 5.73) among underweight smokers. The additive scale interaction was positive for underweight smokers and negative for overweight/obese smokers. Targeted interventions addressing smoking cessation and optimal maternal weight may reduce risks.

## 1. Introduction

Low birthweight (LBW), defined as less than 2500 g (5 pounds, 8 ounces), is often used as a measure of current and future population health in correlation with infant morbidity and mortality, adult chronic disease, mental health, and socioeconomic status [1,2]. The rates of LBW in the United States remain high (8.6%) [1,2,3,4,5]. Between 2014 and 2024, the overall rate of LBW increased more than 6% [6]. LBW and gestational age determine immediate infant survival and potential risk of mortality in their first year of life [6,7]. The current prevalence of LBW among term infants (37–41 weeks gestation) in the U.S. is about 3–4% and slightly increasing [8]. Term infants are likely to follow the pathway of fetal growth restriction (FGR) and be born small-for-gestational-age (SGA) with a birth weight that falls below the 10th percentile of the recommended birthweight for sex and gestational age [9]. SGA infants can be small due to genetics (constitutionally small) or from a pathological process restricting fetal growth (often due to placental insufficiency). Unlike LBW, which is based on absolute birth weight, SGA reflects birth weight relative to gestational age-specific growth standards [9,10]. About 25–40% of SGA infants born at term (≥37 weeks gestation) are constitutionally small, mature and usually healthy [9,10]. Growth-restricted term SGA infants (at the bottom of the 10th percentile) may weigh 2500 g or more and thus do not meet criteria for LBW [11].

Newborns, who are SGA and LBW, are at a particularly high risk for mortality (e.g., stillbirth, neonatal death), early morbidity (e.g., low Apgar score, seizures, intraventricular hemorrhage, cerebral palsy, hypoxic/ischemic encephalopathy), stunting, neurodevelopmental deficit (e.g., learning disabilities, Attention Deficit Disorder, Autism Spectrum Disorders), and experience a range of chronic diseases in adult life (e.g., respiratory, cardiovascular disease, diabetes, obesity, hypertension) [9,10,11,12,13].

LBW and SGA result from complex interactions between maternal, fetal, and environmental factors [14,15,16,17,18,19,20,21,22]. Defining vulnerability of infants at term based on LBW alone can exclude SGA infants who are small and have normal birth weight (>2500 g), but remain vulnerable. The combined clinical outcome for term SGA/LBW infants is distinct from preterm, and especially concerning because it implies pathological in utero fetal growth restriction (prolonged nutritional or oxygen deprivation), poor infant health, and long-term risks through adulthood. Assessment of joint effects per gestational age can more accurately identify and help manage these infants [9,10,11,12,13].

Maternal pre-pregnancy weight is another independent potentially modifiable risk factor that influences newborn weight at term [23]. The prevalence of obesity among women entering pregnancy has been rising globally. Currently, nearly 1 in 5 women in the U.S. have a body mass index (BMI) of 30 or higher [23,24,25]. High pre-pregnancy BMI increases the odds of both large-for-gestational-age (LGA, birth weight > 90th percentile) and SGA births [26,27]. An estimated 50% or more of pre-pregnancy obese women (BMI ≥ 30) smoke, and many of them are likely to continue smoking during pregnancy [28,29]. Higher maternal BMI level may attenuate the association between smoking and LBW and potentially result in fewer SGA births among heavier women [22,28,29,30,31,32,33,34].

Few studies focus on largely overlooked health risks in LBW and SGA infants born at term (37–41 weeks gestation) in association with risk factors at the start of pregnancy, such as maternal smoking and pre-pregnancy BMI [12,13]. Our study investigates the impact of declining maternal smoking and rapidly rising pre-pregnancy BMI in the U.S. on LBW and SGA infants at term, who face significantly higher vulnerability than their normal size and weight counterparts. Combining these measures captures complex interactions by highlighting the harmful effects of maternal smoking and BMI on SGA and LBW outcomes outside the context of preterm birth. Although gestational weight gain (GWG), gestational hypertension (GHTN), and gestational diabetes (GDM) influence fetal growth and newborn outcomes [35,36], they represent downstream clinical pregnancy complications and are considered potential intermediate pregnancy-related factors. To expose the distinct impacts of maternal smoking and BMI on term SGA and LBW outcomes, we intentionally omitted GWG, GHTN, and GDM as covariates in our analytic models.

## 2. Materials and Methods

### 2.1. Study Sample

**Figure 1:**
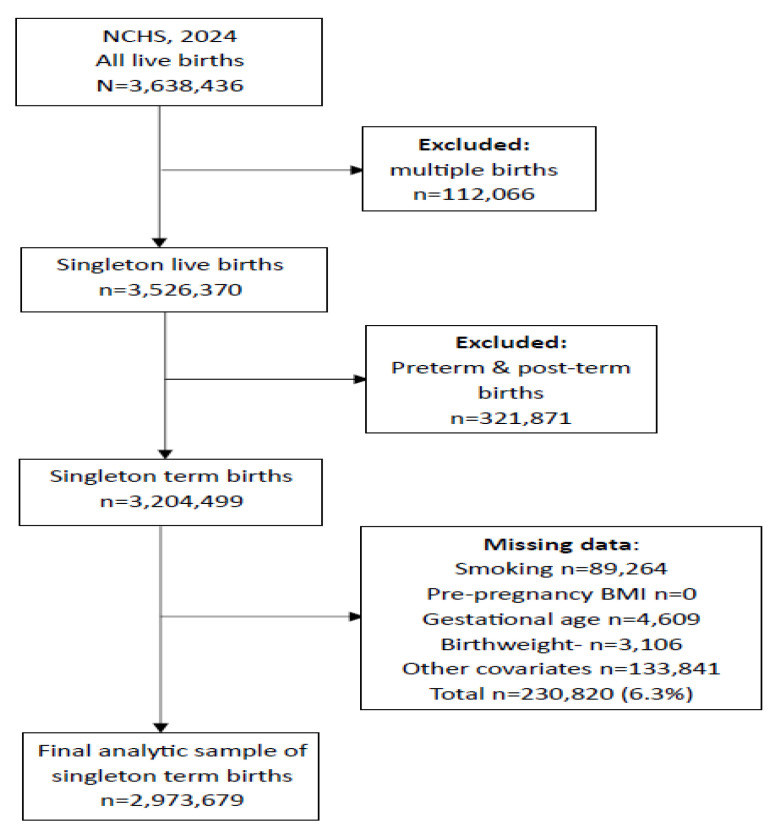
Flow diagram of the study population, 2024 NCHS natality file.

Data of all live births in the U.S. for the year 2024 (N = 3,638,436) were obtained from the U.S. National Center for Health Statistics (NCHS) [37]. The data were first restricted to all singleton live births by excluding multiple-birth data (n = 112,066), then further restricted to singleton term births by excluding preterm and post-term births data (n = 321,871). After deleting missing observations for any covariate, our analyses were limited to complete-case data (n = 2,973,679) of singleton term births, defined as 37 to 41 completed weeks gestation [8] (Figure 1).

### 2.2. Exposure and Outcome Ascertainment

Maternal smoking and pre-pregnancy BMI were the primary exposure variables. Maternal smoking was self-reported and defined as any cigarette use in the three trimesters of pregnancy. Pre-pregnancy BMI was calculated from self-reported maternal height and pre-pregnancy weight (weight in kg divided by height in m^2^) and categorized as underweight (<18.5 kg/m^2^), normal weight (18.5–24.9 kg/m^2^), and combined overweight and obese (≥25 kg/m^2^) categories [38]. Combining overweight and obese BMI categories into a single binary indicator is a common approach in epidemiologic modeling, supported by the established literature, and can be justified by interrelated statistical (maximizing statistical power, simplifying relationships because BMI cut points are largely arbitrary) and biological (continuous pathological mechanisms, similar metabolic profiles of clinical obesity) rationales [39]. Our primary focus is on the association between the currently rising prevalence of maternal pre-pregnancy weight, declining smoking and changing birth outcome distributions, rather than the risk assessment across all distinct BMI categories.

The primary outcomes were low birthweight (LBW), small-for-gestational age (SGA), and combined SGA and LBW (SGA/LBW), applying criteria for term births. LBW was defined as birth weight less than 2500 g [1,2]. Small-for-gestational-age (SGA) was defined as birth weight below the 10th percentile for gestational age and infant sex using externally derived U.S. reference curves developed by Oken et al. and based on national natality data [40]. SGA measurement compares infant birth weight with a national distribution of live births using sex-specific 10th-percentile levels for infants of the same gestational age in weeks. Both birth weight and gestational age in weeks (37–41 weeks) were entered as numerical variables to allow examination of SGA at term.

### 2.3. Covariate Assessment

Covariates were selected a priori based on knowledge, prior literature, and availability in the dataset, and included maternal sociodemographic (race/ethnicity, age, education, marital status, health insurance), reproductive and pregnancy characteristics (parity), and infant sex. Medicaid insurance is a joint federal and state program that provides free or low-cost health coverage to low-income individuals, families, pregnant women, children, the elderly, and people with disabilities in the US. (https://www.medicaid.gov/about-us/program-history/index.html accessed on 30 June 2026). Definitions of gestational weight gain (GWG) are categorized based on a woman’s pre-pregnancy BMI. Adequate GWG is defined as gaining a total amount of weight during pregnancy that falls within the healthy and clinically recommended range established by the Institute of Medicine (IOM), and inadequate or excessive GWG is defined as gaining total weight that falls below or above those specific, recommended thresholds [39].

### 2.4. Statistical Analysis

**Figure 2:**
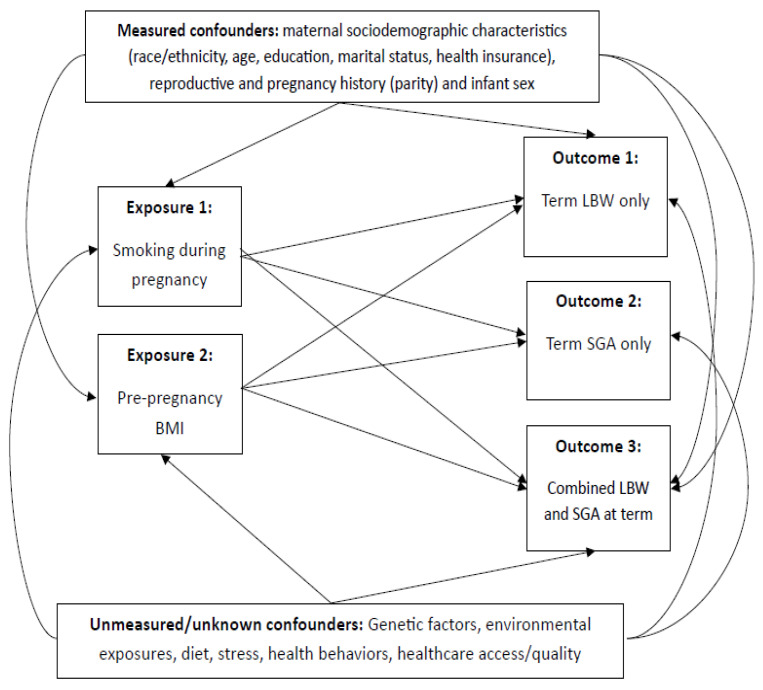
Directed acyclic graph (DAG) for the association between maternal smoking during pregnancy and pre-pregnancy BMI on LBW, SGA, and combined LBW/SGA outcomes.

The prevalence of LBW and SGA among singleton term births in the U.S. in 2024 was examined across maternal and pregnancy characteristics, and the prevalence of smoking during pregnancy was examined according to maternal pre-pregnancy BMI categories. Multivariable logistic regression models were used to estimate independent and joint associations of maternal smoking and pre-pregnancy BMI with term LBW only, term SGA only, and combined term SGA/LBW. Additive interactions were assessed by the relative excess risk due to interaction (RERI) using the formula: [RR11 − RR10 − RR01 + 1] and calculated from the regression estimates, while the corresponding 95% confidence intervals were derived using the delta method [41,42]. Positive RERI values indicate a synergistic interaction, whereas negative values indicate an antagonistic interaction [41,42]. Logistic regression models were adjusted for maternal sociodemographic characteristics (race/ethnicity, age, education, marital status, health insurance), reproductive and pregnancy history (parity), and infant sex. A directed acyclic graph (DAG) was used to visualize the associations and measured and unmeasured confounders (Figure 2). In a sensitivity analysis, the logistic regression models were adjusted for GWG, GHTN, and GDM. All analyses were conducted using SAS version 9.4 (SAS Institute Inc., Cary, NC, USA), and the level of significance was set at α = 0.05. This was a secondary analysis of deidentified and publicly available data, and the study did not require approval by the Institutional Review Board (IRB).

## 3. Results

There were 2,973,679 singleton term births in the U.S. in 2024, of which 2.8% were LBW and 8.1% were SGA (Table 1). A higher prevalence of LBW and SGA deliveries at term was observed among non-Hispanic Black mothers, younger women (<20 years), those with lower educational attainment, unmarried status, and with Medicaid insurance. Likewise, nulliparous women had higher rates compared to multiparous women. Term LBW and SGA varied by pre-pregnancy BMI category, with underweight women exhibiting the highest rates of LBW (6.7%) and SGA (16.9%), while overweight/obese women had the lowest rates (2.4% and 6.8%, respectively). Smoking during pregnancy and inadequate gestational weight gain were strongly associated with increased LBW (7.1% and 4.9%, respectively) and SGA (16.0% and 13.0%, respectively), whereas excessive weight gain was associated with lower rates of LBW and SGA (1.8% and 5.7%, respectively). GHTN was associated with a higher rate of term LBW (4.8%) and SGA (9.1%), while GDM was associated with slightly lower rates (2.6% for LBW and 6.6% for SGA). Cesarean delivery was associated with a modestly higher prevalence of LBW (3.3%), and female infants at term had higher LBW and SGA rates compared to males. Neonatal intensive care unit (NICU) admission was more frequent among SGA (13.6%) than among LBW (8.1%) infants.

In 2024, 2.2% of mothers who delivered a singleton infant at term reported smoking during pregnancy. The smoking prevalence was highest among underweight women (3.9%), followed by overweight/obese (2.3%) and normal weight women (1.9%) (*p* < 0.001) (Table 2). In the adjusted logistic regression models (Table 3), smoking was associated with over 2-fold higher odds of LBW (aOR 2.19; 95% CI: 1.92, 2.51), almost 2-fold higher odds of SGA (aOR 1.90; 95% CI: 1.85, 1.96), and nearly 3-fold increased odds of combined SGA/LBW (aOR 2.75; 95% CI: 2.66, 2.84). Compared to normal BMI, underweight BMI status was associated with higher odds of all adverse birth outcomes, especially SGA/LBW (aOR 1.95; 95% CI: 1.89, 2.01), whereas overweight/obesity status was associated with lower odds, particularly SGA (aOR 0.69; 95% CI: 0.68, 0.70). Analysis of joint effects demonstrated the highest odds of SGA/LBW (aOR 5.14; 95% CI: 4.62, 5.73) among underweight smokers, followed by normal-weight smokers (aOR 3.18; 95% CI: 3.02, 3.34) and overweight/obese smokers (aOR 1.86; 95% CI: 1.77, 1.94) (Table 3). Additive interaction analysis suggested a significant positive (synergistic) interaction between smoking and underweight BMI for combined SGA/LBW (RERI 1.05; 95% CI: 0.78, 1.32), and significant negative (antagonistic) interactions (less-than-additive effects) between smoking and overweight/obesity on SGA (RERI −0.40; 95% CI: −0.52, −0.28) and on SGA/LBW (RERI −1.07; 95% CI: −1.30, −0.85). In our sensitivity analyses, adjusting for GWG, GHTN, and GDM did not change the observed associations, and the primary odds ratios (aORs) and corresponding 95% confidence intervals (CIs) for maternal smoking and BMI remained stable. This lack of attenuation indicates that the observed associations are not confounded by complications occurring during pregnancy.

## 4. Discussion

In this nationally representative study of nearly 3 million singleton term births, both maternal smoking during pregnancy and pre-pregnancy BMI were independently and jointly associated with the odds of LBW and SGA infants. Several important findings emerged. First, smoking during pregnancy remained strongly and consistently associated with newborn outcomes, with more than twofold higher odds of LBW and substantially higher odds of SGA and SGA/LBW. Second, pre-pregnancy BMI demonstrated a graded association with fetal growth measures, with the strongest associations observed among underweight individuals and inverse associations observed among overweight/obese categories, particularly for SGA. Third, the analysis of joint effects revealed marked heterogeneity by BMI category. Underweight smokers had the highest risks for LBW, SGA, and SGA/LBW, and a positive interaction on the additive scale was evident for SGA/LBW.

Consistent with prior and more recent research, we observed disparities in LBW and SGA by sociodemographic characteristics, including higher prevalence among non-Hispanic Black mothers, younger women, those with lower educational attainment, unmarried status, and on Medicaid insurance. These patterns likely reflect broader structural and social determinants of health, including access to care, stress, and environmental exposures [43]. Research strictly confirms that no safe limit of cigarette use exists during pregnancy, and smoking even one cigarette per day harms fetal development [44,45]. Some women, instead of quitting smoking, reduce their smoking intensity during pregnancy, assuming it will decrease health risks in newborns [46,47]. Still, reduction in cigarette consumption does not match the benefit of no smoking. Contemporary meta-analyses and global studies continue to demonstrate that maternal smoking is associated with substantially increased risk of LBW and SGA, often exceeding a two-fold increase in the odds [48]. In our study, the magnitude of association for combined SGA/LBW (aOR 2.75) underscores the compounded vulnerability of term infants exposed in utero to maternal smoking. Despite a relatively low prevalence of smoking among women who delivered a singleton term infant (2.2%), a disproportionate impact on fetal growth highlights its continued public health relevance.

Regarding pre-pregnancy BMI status, underweight BMI was associated with substantially higher odds of LBW and SGA, reflecting inadequate maternal nutritional reserves and suboptimal intrauterine environments [49]. In contrast, overweight/obese BMI was associated with lower odds of SGA or LBW and increased risk for LGA/macrosomia due to greater energy availability [50]. These lower odds of SGA/LBW reflect differences in fetal growth distribution (i.e., shifting infant birth weight upward and increasing LGA/macrosomia births), rather than a beneficial effect of higher maternal BMI [51]. Underweight smokers had more than five-fold higher odds of combined SGA/LBW, compared to normal-weight nonsmokers, suggesting a particularly high-risk subgroup.

Epidemiologic studies support differential effects of smoking across BMI strata, suggesting that the magnitude of smoking-related fetal growth restriction varies by maternal BMI [28,29,30,31,32,33]. In our study, the additive scale interaction indicated a positive RERI for combined SGA/LBW among underweight smokers and a negative RERI for overweight/obese smokers. Biologically, this indicates the convergence of two pathways, nutritional deprivation/excess and toxic exposure attenuation, leading to impairment of placental function and fetal growth [52]. This is in line with other recent studies, where higher maternal BMI appears to partially offset the relative impact of smoking on birthweight [53]. Maternal smoking is strongly associated with a reduction in birth weight and with a significant increase in combined SGA/LBW. Negative impact of nicotine on fetal weight persists across all BMI categories, even as obesity introduces its own set of growth complications [53].

Interaction between declining maternal smoking rates and rising BMI creates a compounding effect and a potentially new pathway for fetal growth measures across term births. [28,29,30,31,32,33]. Term LBW (<2500 g) is largely a subset of growth-restricted SGA births, and reduced rate of SGA leads to a decline in term LBW (fewer growth-restricted infants). Overweight/obese BMI partially buffers the growth-restricting effects of smoking in infants at term (fewer growth-restricted infants and more larger infants) [53]. Nevertheless, this does not imply that smoking is safe in this population; rather, it highlights the complexity of interacting maternal factors and the need to consider effect modification in risk assessment.

Variables that develop post-conception and during pregnancy, such as GWG, GHTN, and GDM, are potential intermediate factors between our baseline exposures and neonatal outcomes, and were intentionally excluded from the primary adjusted logistic regression models to prevent overadjustment bias [54,55]. In our sensitivity analysis, inclusion of GWG, GHTN, and GDM in the regression models yielded virtually no change in the effect sizes of our primary estimates. Given the large study sample, stable odds ratios, and narrow confidence intervals, the empirical evidence is robust and suggests that biological mechanisms of maternal smoking and pre-pregnancy BMI operate along separate pathways, exerting independent impacts on term SGA and LBW. This additionally supports our decision to omit them from the primary analytic models.

This study has several strengths, including a large, nationally representative sample size, availability of numerous variables, and the ability to examine interaction effects with sufficient statistical power. Nevertheless, there are limitations inherent to birth certificate data. Maternal smoking status and pre-pregnancy BMI are self-reported and may be subject to underreporting, potentially biasing associations toward the null. Birth certificates lack detailed data on some important confounders (e.g., diet, nutrition, alcohol, drug use) and obstetric history (e.g., prior preterm birth, LBW delivery). Potential errors in gestational age and outcome classification may also exist. Additionally, BMI does not distinguish between body composition or fat distribution, which may have differing effects on pregnancy outcomes. Complete-case analysis was performed under the assumption that data missingness did not substantially alter exposure–outcome associations [56]. The proportion of missing observations in our study (Figure 1) was relatively low (~6%), reducing concerns regarding major selection bias, yet the potential for selection bias cannot be completely excluded. Lastly, although the robust associations observed between maternal smoking, pre-pregnancy BMI, and term SGA/LBW outcomes underscore critical public health targets, these relationships should not be interpreted as evidence of causal effects in this cross-sectional analysis. Further research utilizing longitudinal data is required to account for any unmeasured variables and to clarify the true directional nature of these relationships.

## 5. Conclusions

This study demonstrates significant, independent relationships between declining maternal smoking, rising pre-pregnancy BMI, and term SGA or LBW outcomes. The strength of these associations highlights a critical modifiable window in preconception for early prenatal intervention. Addressing these maternal risk factors through targeted clinical screening and public health initiatives is essential to reducing the global burden of SGA and LBW and a potential increase in chronic health issues later in life.

## Figures and Tables

**Table 1:** Current low birthweight (LBW) and small for gestational age (SGA) outcomes among singleton term births, 2024 US national sample.

Characteristics	All Term Births	Term LBW	Term SGA
Total N (%)	2,973,679 (100)	82,100 (2.8)	239,746 (8.1)
Maternal Characteristics			
Race/ethnicity			
Non-Hispanic White	1,502,609 (50.5)	30,764 (2.0)	90,462 (6.0)
Non-Hispanic Black	367,024 (12.4)	20,159 (5.5)	51,274 (14.0)
Hispanic	812,572 (27.3)	20,326 (2.5)	65,549 (8.1)
Other race/ethnicity	291,474 (9.8)	10,851 (3.7)	32,468 (11.1)
Age, years			
<20	113,359 (3.8)	5293 (4.7)	15,306 (13.5)
20–34	2,244,590 (75.5)	60,462 (2.7)	179,732 (8.0)
≥35	615,730 (20.7)	16,345 (2.6)	44,715 (7.3)
Education level			
High school or less	1,119,586 (37.6)	38,995 (3.5)	109,717 (9.8)
Some college or higher	1,854,093 (62.4)	43,105 (2.3)	130,036 (7.0)
Marital status			
Married	1,630,612 (54.8)	35,149 (2.2)	106,495 (6.5)
Unmarried	1,343,067 (45.2)	46,951 (3.5)	133,258 (9.9)
Health insurance			
Medicaid	1,162,933 (39.1)	40,761 (3.5)	114,452 (9.8)
Private	1,571,378 (52.8)	35,704 (2.3)	107,052 (6.8)
Self-pay/other	239,368 (8.1)	5,635 (2.4)	18,249 (7.6)
Parity			
First	1,208,581 (40.6)	41,997 (3.5)	125,675 (10.4)
Second or more (Multiparous)	1,765,098 (59.4)	40,103 (2.3)	114,078 (6.5)
Pregnancy Characteristics			
Pre-pregnancy BMI			
Underweight (<18.5)	76,290 (2.6)	5101 (6.7)	12,887 (16.9)
Normal weight (18.5–24.9)	1,122,084 (37.7)	34,423 (3.1)	105,299 (9.4)
Overweight/obese (≥25.0)	1,775,305 (59.7)	42,576 (2.4)	121,567 (6.8)
Pre-pregnancy BMI, mean ±SD	27.9 ± 7.7	26.9 ± 7.9	26.7 ± 7.7
Smoking during pregnancy			
Yes	65,218 (2.2)	4,620 (7.1)	10,425 (16.0)
No	2,908,461 (97.8)	77,480 (2.7)	229,328 (7.9)
Prenatal care utilization	2,920,175 (98.2)	79,865 (2.7)	233,566 (8.0)
Gestational weight gain (GWG)			
Inadequate	603,363 (20.3)	29,530 (4.9)	78,565 (13.0)
Adequate	908,375 (30.5)	25,626 (2.8)	78,447 (8.6)
Excessive	1,461,941 (49.2)	26,944 (1.8)	82,741 (5.7)
GWG, mean ± SD	29.6 ± 15.3	24.8 ± 14.3	25.7 ± 14.2
Gestational diabetes	243,413 (8.2)	6327 (2.6)	16,130 (6.6)
Gestational hypertension	276,766 (9.3)	13,255 (4.8)	25,224 (9.1)
Mode of delivery			
Vaginal	2,012,167 (67.7)	50,952 (2.5)	160,816 (8.0)
Cesarean	876,243 (29.4)	28,792 (3.3)	70,564 (8.1)
Other	85,269 (2.9)	2356 (2.8)	8373 (9.8)
Infant sex			
Male	1,511,614 (50.8)	32,258 (2.1)	119,946 (7.9)
Female	1,462,065 (49.2)	49,842 (3.4)	119,807 (8.2)
NICU admission	146,116 (4.9)	11,860 (8.1)	19,821 (13.6)

**Table 2:** Smoking during pregnancy according to maternal pre-pregnancy BMI categories.

Pre-Pregnancy BMI	Smoking n = 65,218 (2.2%)	Nonsmoking n = 2,908,461 (97.8%)	*p*-Value
Underweight (<18.5)	2987 (3.9)	73,303 (96.1)	<0.001
Normal weight (18.5–24.9)	21,784 (1.9)	1,100,300 (98.1)	
Overweight/obese (≥25.0)	40,447 (2.3)	1,734,858 (97.7)	

**Table 3:** Logistic regression models for independent and joint effects of maternal smoking and pre-pregnancy BMI on term LBW, SGA, and combined SGA/LBW, and the additive interaction using the relative excess risk due to interaction (RERI).

Exposure	Categories	LBW Only n = 5147 (0.2%) aOR (95% CI)	SGA Only n = 162,800 (5.5%) aOR (95% CI)	Combined SGA/LBW n = 76,953 (2.6%) aOR (95% CI)
Independent effects				
Smoking	No (ref.)	1.00	1.00	1.00
	Yes	2.19 (1.92, 2.51)	1.90 (1.85, 1.96)	2.75 (2.66, 2.84)
Pre-pregnancy BMI	Normal (ref.)	1.00	1.00	1.00
	Underweight	1.78 (1.56, 2.02)	1.47 (1.43, 1.51)	1.95 (1.89, 2.01)
	Overweight/obese	0.80 (0.75, 0.85)	0.69 (0.68, 0.70)	0.72 (0.71, 0.73)
Joint effects				
Smoking	BMI categories			
No	Normal (ref.)	1.00	1.00	1.00
No	Underweight	1.76 (1.54, 2.01)	1.47 (1.44, 1.52)	1.92 (1.85, 1.98)
No	Overweight/obese	0.83 (0.78, 0.88)	0.68 (0.67, 0.69)	0.75 (0.74, 0.76)
Yes	Normal	2.23 (1.80, 2.77)	1.99 (1.91, 2.08)	3.18 (3.02, 3.34)
Yes	Underweight	3.22 (2.02, 5.15)	2.56 (2.30, 2.83)	5.14 (4.62, 5.73)
Yes	Overweight/obese	1.82 (1.53, 2.17)	1.28 (1.23, 1.33)	1.86 (1.77, 1.94)
Additive interaction				
Interaction terms		LBW only RERI (95% CI)	SGA only RERI (95% CI)	SGA/LBW RERI (95% CI)
Smoking × underweight vs. normal BMI		0.23 (−0.82, 1.29)	0.09 (−0.06, 0.23)	1.05 (0.78, 1.32)
Smoking × overweight/obese vs. normal BMI		−0.24 (−1.13, 0.67)	−0.40 (−0.52, −0.28)	−1.07 (−1.30, −0.85)

aOR: adjusted odds ratio; CI: confidence interval; Adjusted for: race/ethnicity, age, education, marital status, health insurance, parity, and infant sex; RERI: relative excess risk due to interaction = RR_11_ − RR_10_ − RR_01_ + 1.

## Data Availability

Public-use data in this study were deidentified and are available for download via the Vital Statistics Online Data Portal at https://www.cdc.gov/nchs/data_access/vitalstatsonline.htm Accessed on 6 April 2026.
